# Ruminal methanogen abundance and associated physiological characteristics in Japanese Black–Holstein F1 calves

**DOI:** 10.5713/ab.260205

**Published:** 2026-07-27

**Authors:** Huseong Lee, Yuta Suetomi, Satoshi Haga, Sanggun Roh

**Affiliations:** 1 Graduate School of Agricultural Science, Tohoku University, Sendai, Japan; 2 Institute of Agriculture, Academic Assembly, Shinshu University, Nagano, Japan; 3 Cattle and Swine Technical Section Research Laboratory, Itochu Feed Mills Corporation, Nasushiobara, Japan

**Keywords:** Blood Metabolites, Methanogenic Archaea, Rumen Fermentation, Young Ruminant

## Abstract

**Objective:**

This study investigated associations among ruminal total methanogen abundance, rumen microbiota, fermentation characteristics, feed intake, growth performance, and blood biochemical parameters in Japanese Black–Holstein F1 calves during early life.

**Methods:**

Twenty-three male Japanese Black–Holstein F1 calves were raised as a single cohort under identical feeding conditions from one to nine weeks old. All calves were fed the same milk replacer until weaning and had *ad libitum* access to starter and hay throughout the trial. Based on ruminal total methanogen abundance measured by quantitative real-time polymerase chain reaction at the end of the experiment, calves in the upper and lower quartiles were retrospectively classified as high- and low-methanogen groups, respectively (n = 6 per group). Rumen microbiota was characterized using 16S rRNA gene amplicon sequencing. Growth performance, feed intake, rumen fermentation characteristics, blood biochemical parameters, and rumen microbial profiles were compared between groups, and correlations with total methanogen abundance were assessed using all calves.

**Results:**

Total methanogen abundance was higher in the high-methanogen group compared to the low-methanogen group (6.56 vs. 5.53 log copy number/ng DNA; p<0.001). Average daily gain did not differ between groups (0.64 vs. 0.69 kg/d). Starter intake was higher in the high-methanogen group at 6 weeks (p<0.05) and was positively correlated with total methanogen abundance (r = 0.42; p<0.05). Molar percentage of ruminal propionate was higher in the low-methanogen group (28.59% vs. 20.58%; p<0.05). *Methanobrevibacter* was enriched in the high-methanogen group (p<0.05), whereas *Prevotella* (p<0.01), *Succinivibrio* (p<0.05), and *Selenomonas* (p<0.05) were enriched in the low-methanogen group.

**Conclusion:**

These findings suggest that ruminal total methanogen abundance during early life is related with distinct rumen fermentation patterns and microbial signatures, highlighting potential microbial and nutritional factors linked to methanogen abundance in calves.

## INTRODUCTION

Methane emissions from agriculture are a substantial component of anthropogenic greenhouse gases, and enteric fermentation in ruminants is a major contributor. Methane is a potent greenhouse gas, with a global warming potential 28 times greater than that of carbon dioxide. In the rumen, methane is produced exclusively by methanogenic archaea as a byproduct of anaerobic microbial fermentation [1]. This methane production is not only an environmental concern but also represents a loss of 2%–12% of the animal’s ingested energy [2], making methane reduction a critical topic not only for environmental sustainability but also for improving production efficiency.

Various interventions, such as feed additives, antibiotic use, and total rumen content exchange, have been attempted to suppress methane production. Feed strategies aiming to reduce methane have included the use of 3-nitrooxypropanol, saponins, tannins, nitrates, and unsaturated fatty acids. However, in most cases, the microbial community tends to revert to its original state after treatment cessation, making it difficult to achieve lasting effects [3]. This limitation highlights the need to better understand the biological factors underlying natural variation in methane-related traits among animals. Moreover, considerable inter-individual variation in methane emissions has been reported even under the same feeding conditions [4–6], suggesting that differences in rumen microbial ecology and host-related physiological traits may contribute to individual variation in methane-related phenotypes. Therefore, identifying microbial and physiological characteristics associated with methanogen abundance during early rumen development may help clarify why calves differ in rumen fermentation patterns under similar management conditions.

The colonization of rumen microbes begins immediately after birth and follows a defined and progressive succession. Notably, cellulolytic bacteria and methanogenic archaea have been detected as early as 2–4 days after birth [7], even before solid feed ingestion. In particular, methanogenic archaea develop progressively from immediately after birth and reach adult-level densities by around 14 days of age. Most of the functional microbial taxa found in adult animals-including key bacterial and archaeal groups-emerge within the first week of life, with continued compositional shifts occurring throughout the suckling and pre-weaning stages [8]. In adult ruminants, the rumen harbors a diverse and mature microbial community that is typically more resistant and resilient to disturbances compared with less developed communities [9]. By contrast, evidence from studies in small ruminants indicates that the rumen microbiota during early life is more plastic and, therefore, potentially more amenable to modulation. Targeted interventions during this period-such as dietary modulation or microbial inoculation-may have enduring effects on rumen function and methane emissions later in life [10–12]. These findings suggest that the calf stage may represent an important period for understanding the establishment of rumen microbial communities, including methanogenic archaea. However, much of the current understanding of methanogen establishment before full rumen development has been derived from studies in lambs or goats, highlighting the need for more targeted research in calves. Moreover, few studies have examined whether naturally occurring differences in methanogen abundance in calves raised under identical feeding conditions are accompanied by differences in rumen fermentation, microbial composition, feed intake, growth performance, and blood biochemical parameters.

Because calves were raised as a single cohort under identical feeding conditions and retrospectively classified based on ruminal total methanogen abundance, this study was designed to evaluate associations rather than causal effects of methanogen status. We hypothesized that calves with higher ruminal total methanogen abundance would show distinct rumen microbial, fermentation, and physiological profiles compared with calves with lower methanogen abundance, even when raised under identical feeding conditions. The objective of this study was to clarify whether naturally occurring differences in ruminal total methanogen abundance are associated with distinct microbial, fermentative, nutritional, and physiological characteristics in calves raised under identical feeding conditions.

## MATERIALS AND METHODS

### Animals

**Figure 1:**
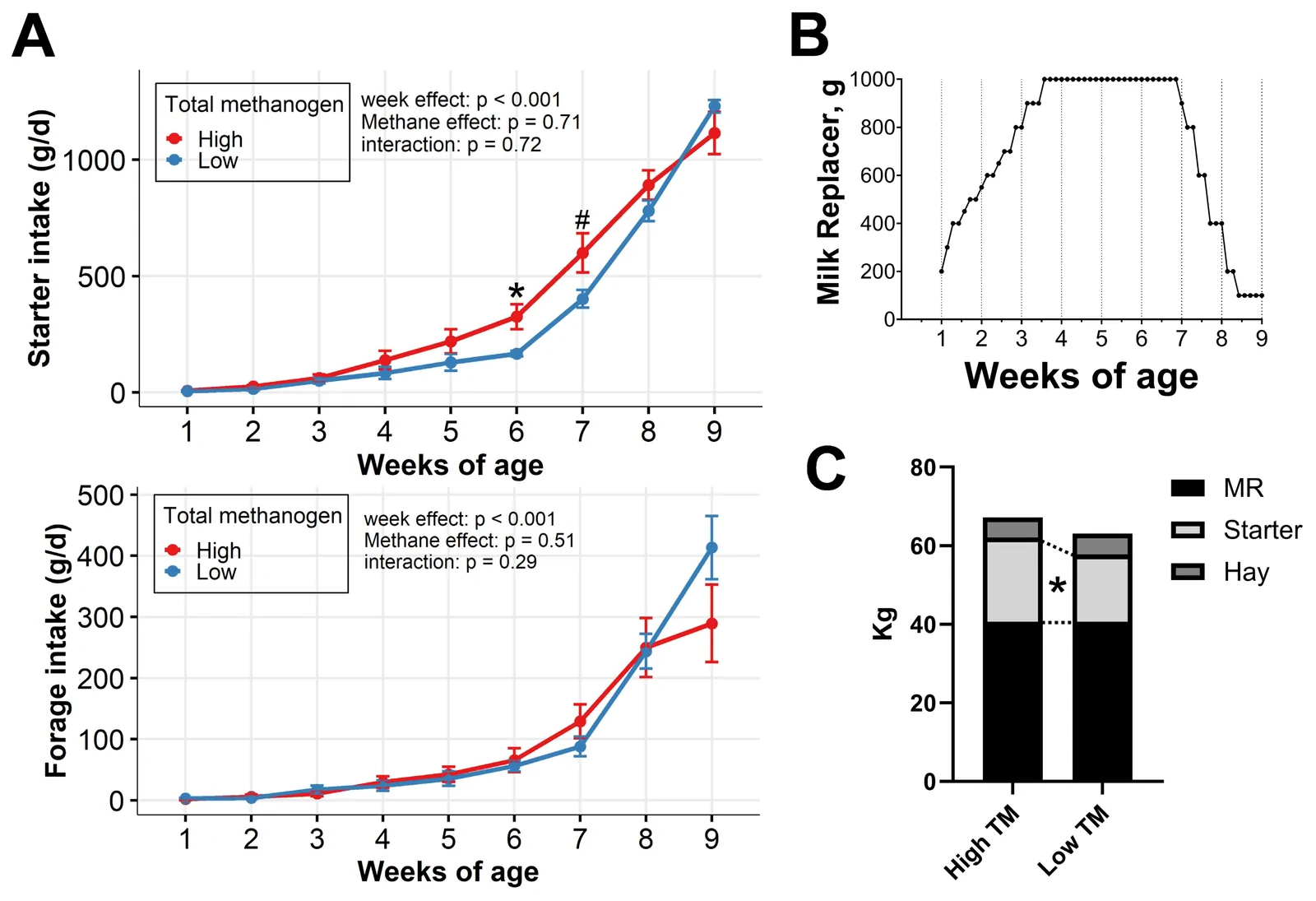
Feed intake patterns and milk replacer-feeding program in calves with high and low ruminal total methanogen abundance during the experimental period. (A) Weekly starter and forage intake, (B) milk replacer-feeding program, and (C) cumulative intake of milk replacer, starter, and hay during the experimental period in calves with high ruminal total methanogen abundance (High TM) and low ruminal total methanogen abundance (Low TM). Calves were classified into the High TM and Low TM groups based on ruminal total methanogen abundance. The same milk replacer-feeding program was applied to all calves. Data are presented as the mean±SEM. p-values indicate the effects of week, TM group, and their interaction. * and ^#^ indicate p<0.05 and 0.05≤p<0.10, respectively. TM, total methanogen; MR, milk replacer; SEM, standard error of the mean.

Twenty-three male Holstein×Japanese Black crossbred (F1) calves, with an initial average body weight of 40.3±8.3 kg, were raised until 9 weeks of age, by which time they reached 80.7±8.5 kg. Milk replacer was administered twice daily (at 9:00 and 15:00), and the same milk replacer-feeding program was applied to all calves. The milk-replacer amount was gradually increased, reaching 1 kg of milk powder per day by day 24. This level was maintained until day 48 and then gradually tapered off until weaning (Figure 1B). Calves had free access to bermudagrass hay, starter feed, and water throughout the experimental period. Weaning was defined as the point when starter intake exceeded 1 kg (as fresh matter) for three consecutive days, which occurred on average at 59.3±3.7 days of age. The intake of both hay and starter was recorded regularly throughout the trial. Growth parameters-including body weight, heart girth, abdominal girth, and wither height-were measured weekly. Following weaning, all calves were given the same diet of starter and bermudagrass hay up to 9 weeks of age. After weaning, all calves continued to receive starter feed and bermudagrass hay until 9 weeks of age. The detailed nutritional composition of the milk replacer, starter, and hay is shown in Table 1. Fecal score was assessed daily by trained observers during milk feeding, using a 4-point scale (1 = normal, 2 = soft, 3 = muddy, 4 = watery).

### Rumen fluid and blood samples collection

Rumen fluid was collected once at the end of the experiment using an oral rumen tube (catalogue no. 16130000; Fujihira Industry). Immediately after collection, the pH was measured using a portable pH meter (LAQUAtwin pH-33B; HORIBA). Samples were then stored at −80°C for subsequent analysis of fermentation parameters and gDNA extraction. Blood samples were taken weekly via jugular vein puncture at 13:00, which was 3 h after the morning milk feeding. Heparinized tubes (Venoject II VP-H100K; Terumo) were used for collection. Samples were centrifuged at 3,000×g for 15 min at 4°C, and the resulting plasma was stored at −30°C for later metabolic analysis.

### Blood metabolites analysis

Blood biochemical parameters were measured using an automatic biochemical analyzer (HITACHI 7070; Hitachi). The following analytes were measured: total protein, albumin, globulin, blood urea nitrogen (BUN), creatinine, total cholesterol, triglycerides, non-esterified fatty acids (NEFA), phospholipids, glucose, beta-hydroxybutyrate (BHBA), alkaline phosphatase (ALP), aspartate aminotransferase (AST), alanine aminotransferase (ALT), lactate dehydrogenase (LD), gamma-glutamyl transferase (γ-GT), creatine kinase (CK), sodium, potassium, chlorine, calcium, phosphorus and magnesium.

### Rumen fermentation characteristics analysis

The total volatile fatty acids (VFA) and their components, including acetic acid, propionic acid, butyric acid, valeric acid, isobutyric acid, and isovaleric acid, were separated and measured by gas chromatography (model 6850; Agilent Technologies) using a capillary column (30 m×0.25 mm×0.25 μm; Agilent J&W GC Columns). The operating conditions were as follows: carrier gas, He; flow rate, 30 mL/min; injector temperature, 160°C; FID detection, 250°C; and column oven temperature, 130°C. The concentration of ammonia nitrogen was determined using the phenol-hypochlorite method. Each 10 μL sample was mixed with 1 mL of phenol color reagent and alkali-hypochlorite reagent. The mixture was incubated at 37°C for 15 min, and the optical density was measured at a wavelength of 630 nm using a spectrophotometer (BioTek).

### Genomic DNA extraction and quantitative real-time polymerase chain reaction

Genomic DNA was extracted from 300 μL of rumen fluid samples using the bead-beating plus column (RBB+C) method [13] and the QIAamp Fast DNA Stool Mini Kit, following the manufacturer’s instructions. The concentration and purity of the extracted DNA were assessed using a Qubit Flex Fluorometer (Thermo Fisher Scientific) for accurate concentration measurement. The mean concentration of extracted DNA was 69.9±22.0 ng/μL. DNA purity was evaluated based on the A260/280 ratio, and only samples with an A260/280 ratio greater than 1.8 were used for quantitative real-time polymerase chain reaction (qPCR) and 16S rRNA gene amplicon sequencing. Quantification of total bacterial and methanogen populations was performed using qPCR, following the protocols described by Denman and McSweeney [14] and Khafipour et al. [15]. The qPCR reactions were carried out using TB Green Premix Ex Taq II (Takara Biomedical Technology) with specific primer sets targeting each microbial group. For total bacteria, the primer pair consisted of a forward primer (5′-CGGCAACGAGCGCAACCC-3′) and a reverse primer (5′-CCATTGTAGCACGTGTGTAGCC-3′) [14]. For total methanogens, the primers used were uniMet-1F (5′-CCGGA GATGGAACCTGAGAC-3′) and uniMet-1R (5′-CGGT CTTGCCCAGCTCTTATTC-3′) [16]. Each qPCR reaction was performed in triplicate in a final volume of 10 μL, consisting of 5 μL of TB Green Premix Ex Taq II, 1 μL of genomic DNA (10 ng/μL), 0.5 μL of each primer, and 3 μL of diethylpyrocarbonate (DEPC)-treated water. The thermal cycling conditions were as follows: initial denaturation at 95°C for 15 min, followed by 30 amplification cycles of denaturation at 98°C for 10 s, annealing at 55°C for 15 s, and extension at 72°C for 60 s. Absolute quantification of gene copies for each microbial group was achieved using standard curves generated from serial tenfold dilutions of plasmid DNA containing the respective target gene sequences. A total of ten standard reference points were established, and the copy numbers were calculated based on the method described by Chen et al. [17]. The copy number of total bacterial and methanogen 16S rRNA genes per 1 ng of DNA was calculated using the equation (QM×C×DV)/S, where QM represents the mean quantified copy number, C denotes the DNA concentration of each sample (ng/μL), DV is the total dilution volume of the extracted DNA (μL) and S indicates the amount of DNA used for quantification (ng). Based on the total methanogen copy number per ng DNA, calves (n = 23) were ranked, and animals in the highest and lowest quartiles (top and bottom 25%) were retrospectively classified into the high (n = 6) and low (n = 6) TM groups, respectively, to maximize the contrast in methanogen abundance. These classifications were used for association-based comparisons and were not experimental treatments. All calves were managed under identical feeding and housing conditions, including the same milk replacer feeding regimen and free access to starter and hay. No significant differences in initial body weight or age were observed between groups prior to classification.

### Amplicon sequencing and bioinformatic analysis of 16S rRNA gene sequence

Microbial community profiling was performed by targeting the V3–V4 region of the 16S rRNA gene using primers 314-F (5′-CCTACGGGNGGCWGCAG-3′) and 805-R (5′-GACTA CHVGGGTATCTAATCC-3′), following Illumina’s 16S Metagenomic Sequencing Library Preparation protocol. Paired-end sequencing (2×300 bp) was conducted on the Illumina MiSeq platform (Illumina). The obtained 16S rRNA amplicon sequencing reads were processed and analyzed using QIIME2. Adapter and primer sequences were trimmed using Cutadapt, and subsequent steps including chimera removal, denoising, quality filtering (Q-score>25), and merging of paired-end reads were performed using the DADA2 plugin. Taxonomic classification was carried out with the q2-feature-classifier plugin, employing a Naive Bayes classifier trained on the SILVA reference database (ver. 138). Amplicon sequence variants (ASVs) were assigned taxonomy accordingly, and those that were unassigned or corresponded to chloroplast or mitochondrial sequences were excluded from downstream analyses. To account for differences in sequencing depth among samples, the ASV table was rarefied to the minimum sequencing depth across samples. Alpha and beta diversity metrics of the rumen microbiota were compared between the high and low TM groups using MicrobiomeAnalyst. The ASV table, formatted as a Biological Observation Matrix (BIOM), was used to compute alpha diversity indices, including Shannon and Simpson indices. Differences in microbial community composition (beta diversity) were visualized using principal coordinate analysis (PCoA) based on the Bray–Curtis dissimilarity matrix.

### Statistical analysis

Repeated-measures two-way ANOVA was conducted to evaluate the effects of group (high vs. low TM) and age in weeks (time) on growth performance, feed intake, and blood biochemical parameters, using GraphPad Prism software (ver. 10.5). Spearman’s correlation analysis was performed in R (ver. 4.4.3) to assess associations between total methanogen abundance, starter feed intake, rumen microbiota, and fermentation parameters (n = 23). Correlations with a coefficient of |r|≥0.4 and a p-value<0.05 were considered significant and were visualized accordingly. Comparisons of differential microbial taxa, alpha diversity indices, rumen fermentation parameters, and gene copy numbers of total bacteria and methanogens between high and low TM groups were performed using Student’s t-test or the Wilcoxon rank-sum test, as appropriate. Beta diversity between the high and low TM groups was assessed using permutational multivariate analysis of variance (PERMANOVA) based on 9,999 permutations, conducted with PAST3 software. Statistical significance was defined as p<0.05, while a tendency toward significance was considered when 0.05<p<0.10.

## RESULTS

### Feed intake and growth performance

**Figure 2:**
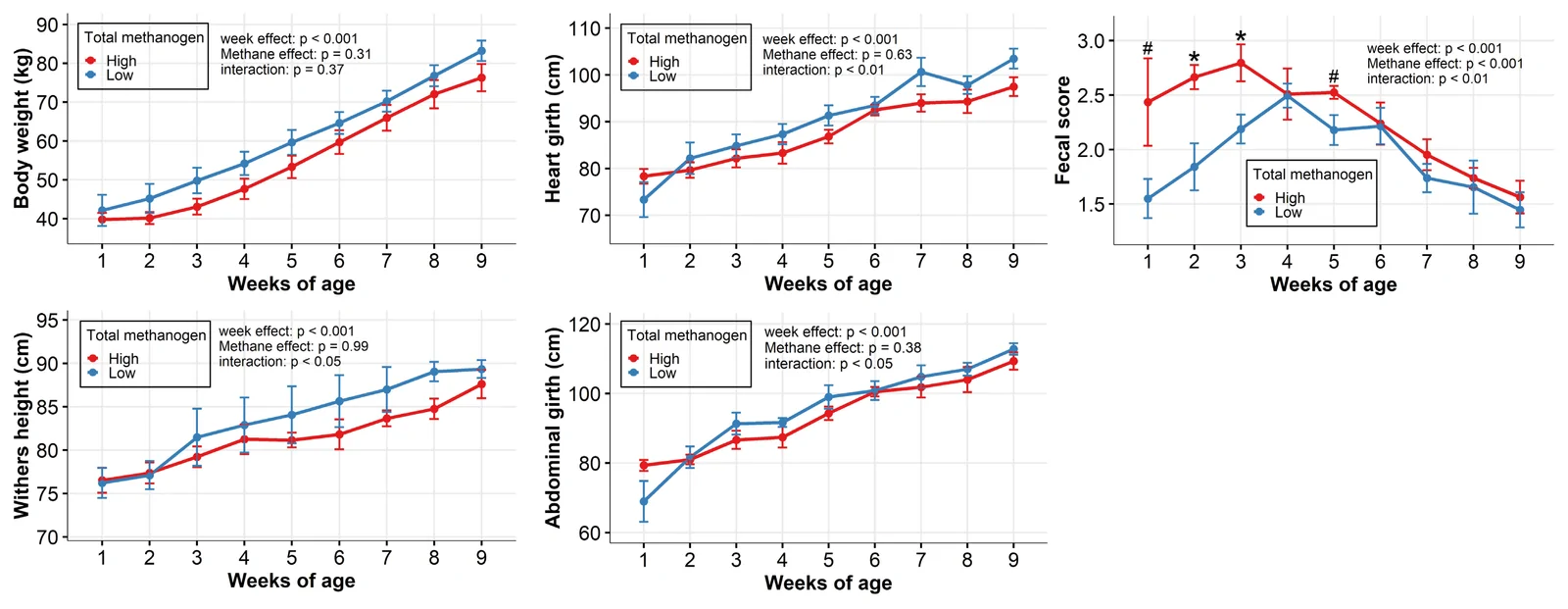
Changes in body weight, heart girth, fecal score, withers height, and abdominal girth in calves with high and low ruminal total methanogen abundance during the experimental period. Body weight, heart girth, fecal score, withers height, and abdominal girth were measured weekly in calves with high ruminal total methanogen abundance (High TM) and low ruminal total methanogen abundance (Low TM). Calves were classified into the High TM and Low TM groups based on ruminal total methanogen abundance. Fecal score was assessed daily by trained observers during milk feeding using a 4-point scale (1 = normal, 2 = soft, 3 = muddy, and 4 = watery), and weekly mean values are shown. Data are presented as the mean±SEM. p-values indicate the effects of week, TM group, and their interaction. * and ^#^ indicate p<0.05 and 0.05≤p<0.10, respectively. TM, total methanogen; SEM, standard error of the mean.

Data for starter feed intake and bermudagrass hay intake are shown in Figure 1A. Starter intake increased with age in both groups, but the increase was more rapid in the high TM group from 6 weeks of age. Starter intake was significantly higher at 6 weeks of age (p<0.05) and tended to be higher at 7 weeks of age (p<0.1). Total starter intake was also significantly higher in the high TM group (p<0.05). Body weight, heart girth, abdominal girth, wither height, and fecal score measurements are presented in Figure 2. Throughout the experimental period, no significant differences in growth performance were observed between the high and low TM groups for any measurement. Fecal score was significantly higher (p<0.05) in the high TM group than in the low TM group at 2 and 3 weeks of age.

### Rumen fermentation and microbial characteristics

A comparison of total methanogen and total bacterial amounts, as well as rumen fermentation characteristics is shown in Table 2. Total methanogen abundance was higher (p<0.0001) in the high TM group than in the low TM group. Ruminal propionate proportion was higher in the low TM group than in the high TM group (p<0.05), whereas butyrate proportion tended to be higher in the high TM group than in the low TM group (p = 0.074).

**Figure 3:**
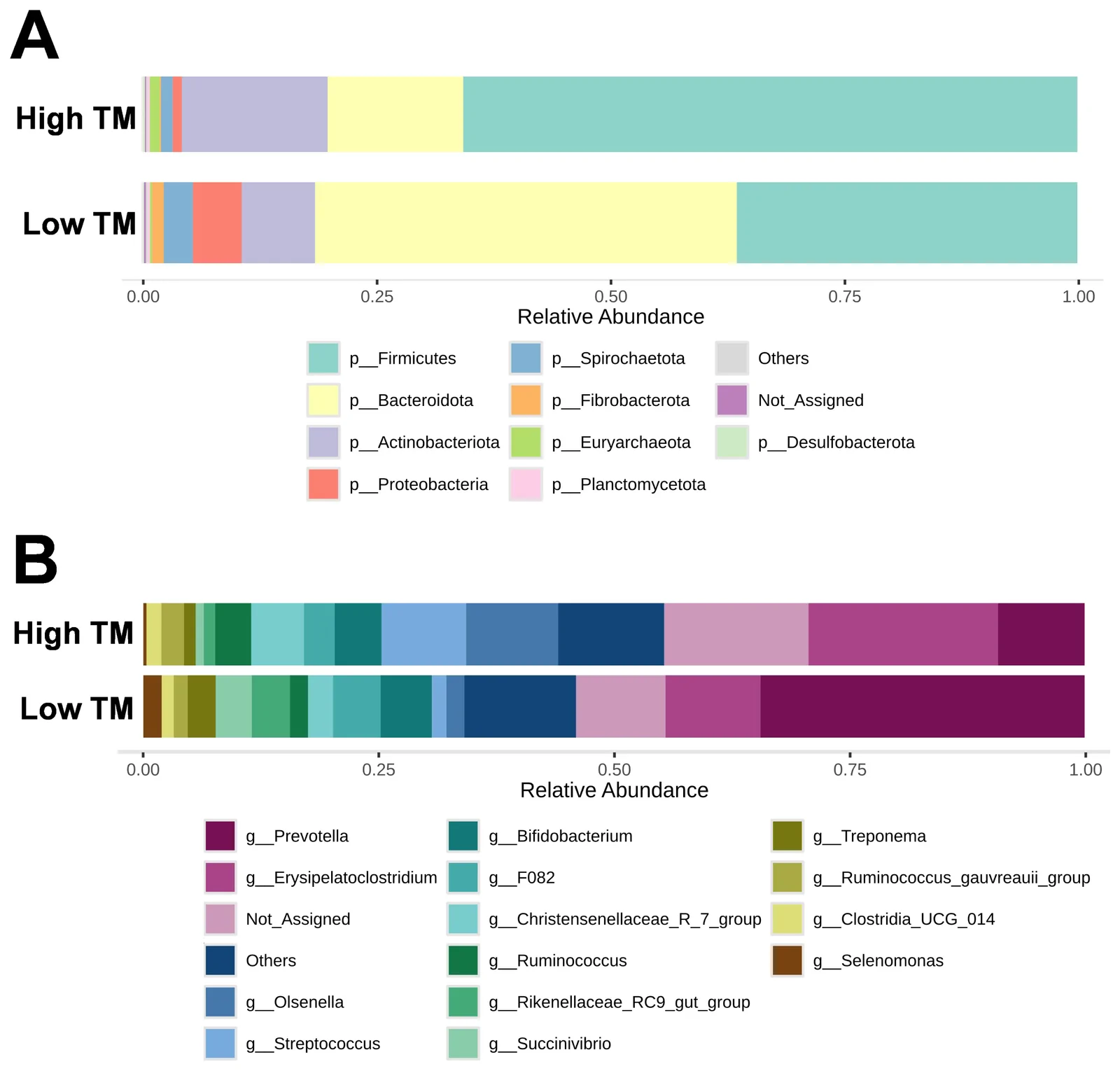
Rumen microbiota composition in calves with high and low ruminal total methanogen abundance. Relative abundances of the (A) top 10 phyla and (B) top 15 genera are shown for calves with high ruminal total methanogen abundance (High TM) and low ruminal total methanogen abundance (Low TM). Calves were classified into the High TM and Low TM groups based on ruminal total methanogen abundance. Others indicates taxa not included in the top 10 phyla or top 15 genera. Not_Assigned indicates taxa that could not be assigned at the corresponding taxonomic level. TM, total methanogen; p_, phylum; g_, genus.

Sequencing of 16S rRNA genes in rumen fluid from F1 calves yielded 1,136,388 raw sequence reads, averaging 49,408±240 reads per sample. Firmicutes were most prevalent, accounting for 56.9% of all sequences across 23 samples, followed by Bacteroidota (23.7%), Actinobacteriota (14%), Proteobacteria (2.1%), Spirochaetota (1.7%), and Euryarchaeota (2.3%). At the genus level, *Erysipelatoclostridium* was dominant (19.3%), followed by *Prevotella* (16.7%), *Bifidobacterium* (8.2%), *Olsenella* (5.3%), Streptococcus (5.2%), and *Ruminococcus* (3.6%). The rumen microbiota profiles at the phylum and genus levels in the high and low TM groups are shown in Figure 3.

**Figure 4:**
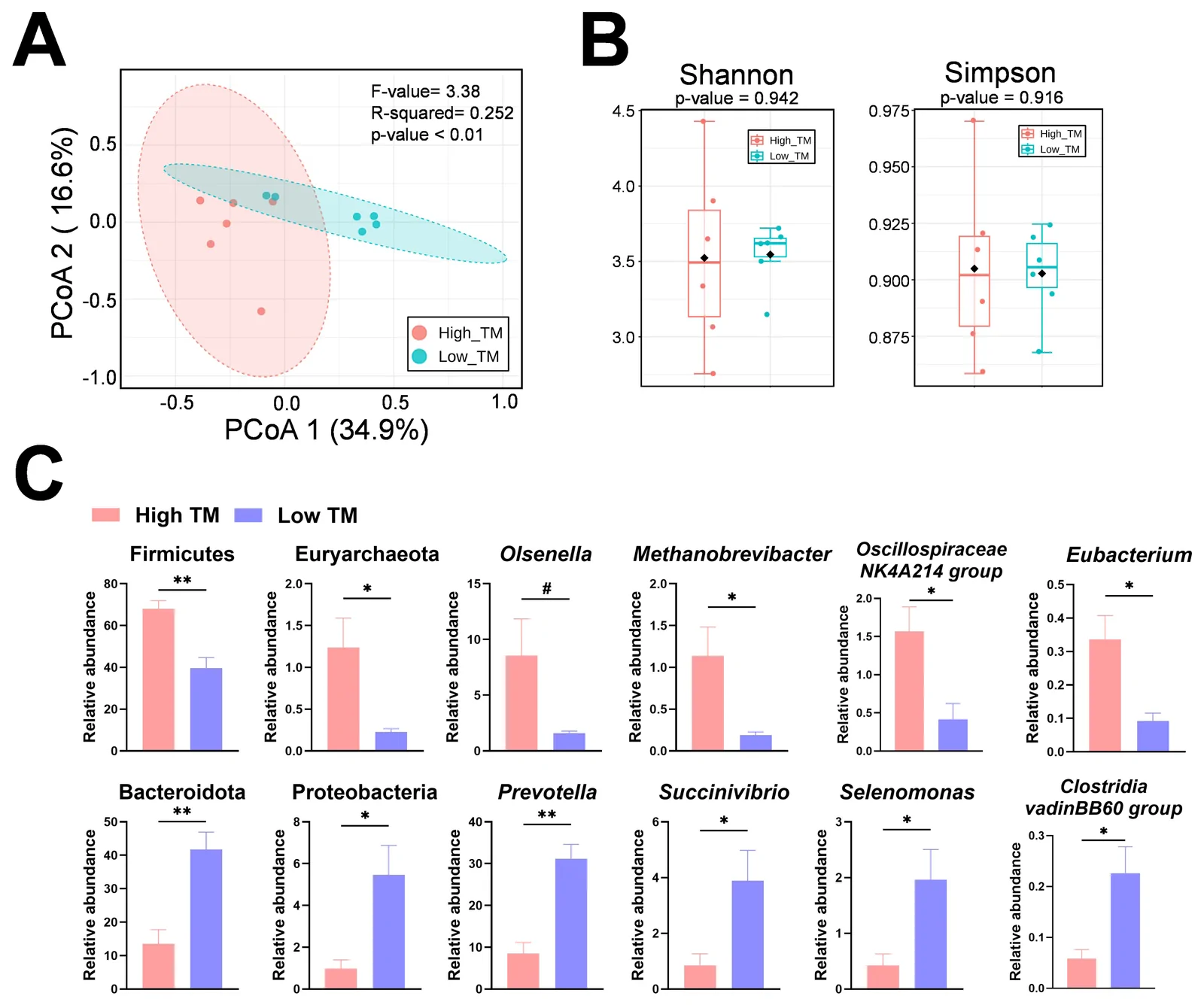
Comparison of rumen microbial community structure, α-diversity, and differentially abundant taxa between calves with high and low ruminal total methanogen abundance. (A) Principal coordinate analysis (PCoA) plot based on the Bray–Curtis dissimilarity matrix, (B) α-diversity indices, including Shannon and Simpson indices, and (C) differentially abundant rumen microbiota between calves with high ruminal total methanogen abundance (High TM) and low ruminal total methanogen abundance (Low TM). Calves were classified into the High TM and Low TM groups based on ruminal total methanogen abundance. Data are presented as the mean±SEM. *, **, and ^#^ indicate p<0.05, p<0.01, and 0.05≤p<0.10, respectively. TM, total methanogen; SEM, standard error of the mean.

PERMANOVA based on Bray–Curtis dissimilarity indicated that rumen microbial community composition differed between the high and low TM groups (p<0.01; Figure 4A). Alpha diversity analysis revealed no significant differences in the Shannon and Simpson indices between the high and low TM groups (Figure 4B). Differentially abundant phyla and genera between the high and low TM groups are shown in Figure 4C. At the phylum level, Firmicutes (p<0.01) and Euryarchaeota (p<0.05) were significantly enriched in the high TM group compared with the low TM group. In contrast, Bacteroidota (p<0.01), and Proteobacteria (p<0.05) were more abundant in the low TM group. At the genus level, *Olsenella* showed a trend toward higher abundance in the high TM group. *Methanobrevibacter* (p<0.05), *Oscillospiraceae_NK4A214_group* (p<0.05), and Eubacterium (p<0.05) were significantly enriched in the high TM group. On the other hand, Prevotella (p<0.01), *Succinivibrio* (p<0.05), *Selenomonas* (p<0.05), and *Clostridia_vadinBB60_group* (p<0.05) were more abundant in the low TM group.

### Correlation analysis of ruminal parameters

**Figure 5:**
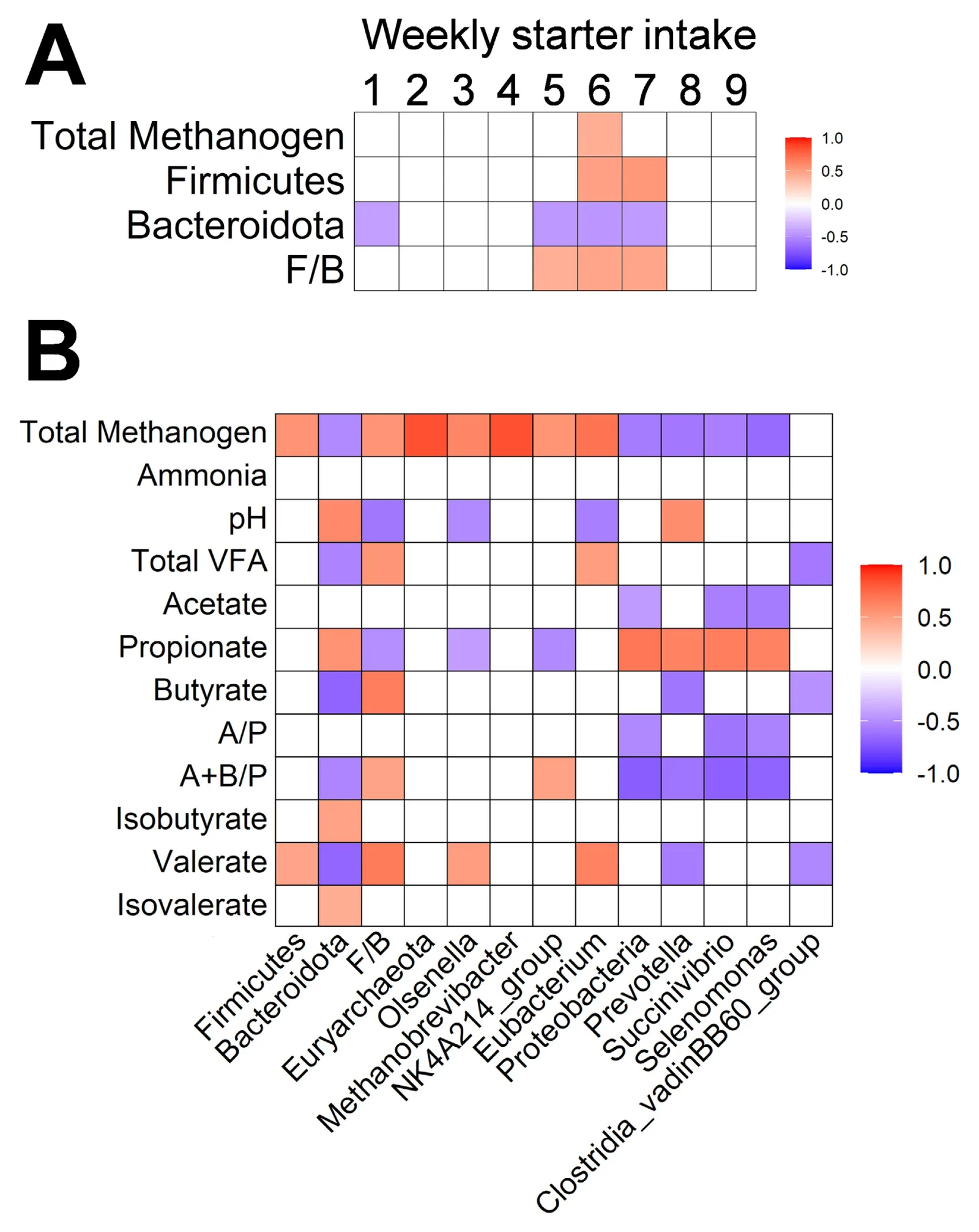
Correlations between differentially abundant rumen microbiota and feed intake or rumen fermentation parameters. (A) Spearman’s correlation heatmap between differentially abundant rumen microbiota and weekly starter intake, and (B) Spearman’s correlation heatmap between differentially abundant rumen microbiota and rumen fermentation parameters (n = 23). Only strong correlations with |r|≥0.4 and p<0.05 are shown. Blank cells indicate correlations that did not meet these criteria. Red and blue indicate positive and negative correlation coefficients, respectively, and color intensity represents the strength of the correlation. F/B, Firmicutes-to-Bacteroidota ratio; VFA, volatile fatty acids; A/P, acetate-to-propionate ratio; A+B/P, acetate plus butyrate-to-propionate ratio.

Spearman’s correlations among weekly starter intake, differentially abundant taxa and rumen fermentation characteristics were analyzed using all samples (n = 23) and are shown in Figure 5 (correlation coefficient, |r|≥0.4; p<0.05). Total methanogen abundance was positively correlated (r = 0.42) with starter intake at 6 weeks of age. The Firmicutes-to-Bacteroidota (F/B) ratio was positively correlated with starter intake at 5 (r = 0.42), 6 (r = 0.47), and 7 (r = 0.46) weeks of age. Methanobrevibacter showed a strong positive correlation (r = 0.84) with total methanogen. The ruminal propionate concentration showed positive correlations with Prevotella (r = 0.63), Succinivibrio (r = 0.65), and Selenomonas (r = 0.63). These genera showed negative correlations (p<0.05) with total methanogen abundance.

### Blood biochemical parameters between high and low TM groups

**Figure 6:**
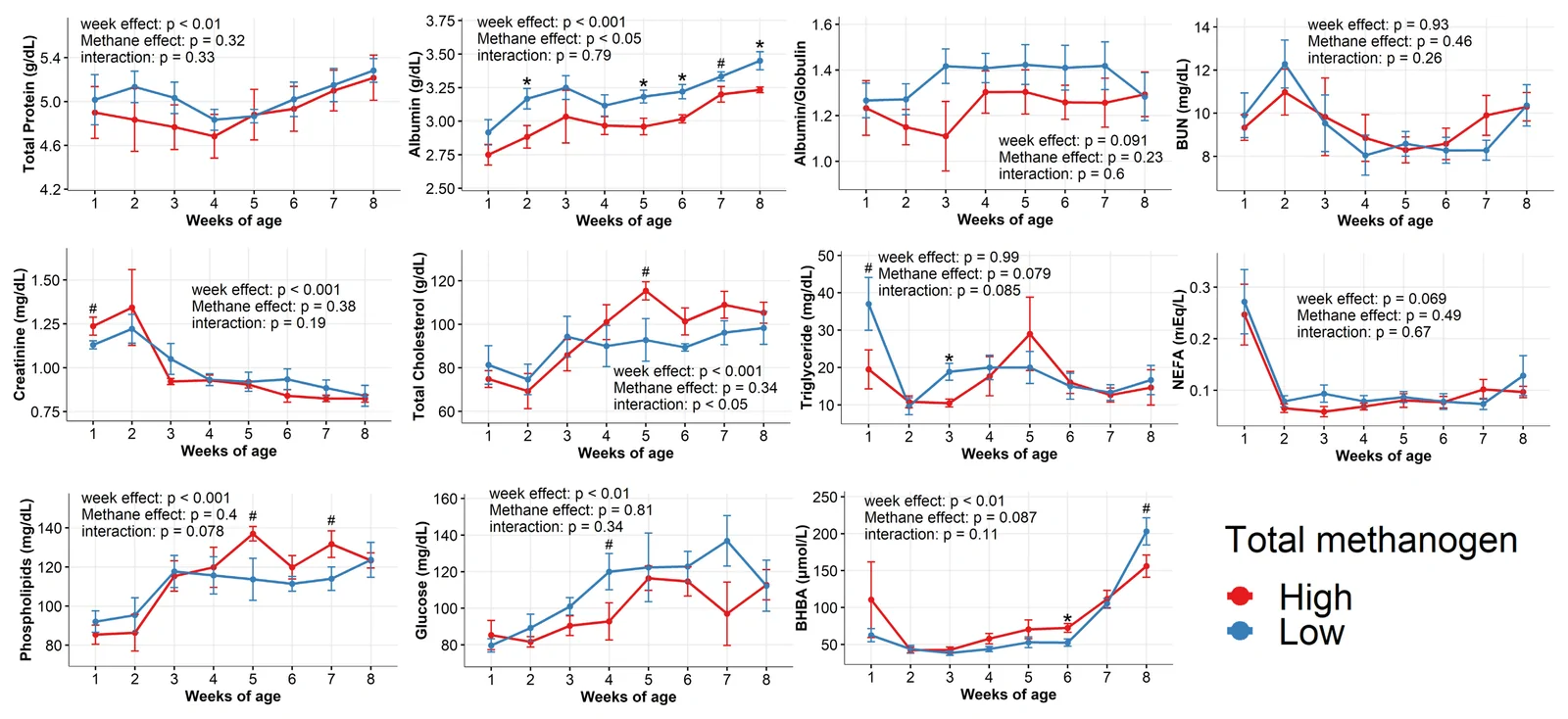
Changes in blood biochemical parameters related to protein, lipid, and energy metabolism in calves with high and low ruminal total methanogen abundance during the experimental period. Blood biochemical parameters were compared between calves with high ruminal total methanogen abundance (High TM) and low ruminal total methanogen abundance (Low TM). Calves were classified into the High TM and Low TM groups based on ruminal total methanogen abundance. Data are presented as the mean±SEM. p-values indicate the effects of week, TM group (Methane effect), and their interaction. * and ^#^ indicate p<0.05 and 0.05≤p<0.10 between groups at each week, respectively. BUN, blood urea nitrogen; NEFA, non-esterified fatty acids; BHBA, β-hydroxybutyrate; TM, total methanogen; SEM, standard error of the mean.

Blood biochemical parameters are presented in Figure 6 and Supplement 1. Across the first 8 weeks of age, most blood biochemical parameters and enzyme activities showed significant age-related changes, whereas group-related differences were observed only in a limited subset of variables. For most parameters, including total protein, A/G ratio, BUN, creatinine, glucose, NEFAs, and major liver and muscle enzymes, no significant main effect of TM group or TM group×week interaction was observed.

Albumin increased with age (week effect, p<0.001) and differed between TM groups (TM group effect, p<0.05), with higher concentrations in the low TM group than in the high TM group. Total cholesterol showed a significant week effect (p<0.001) and TM group×week interaction (p<0.05), but no significant main effect of TM group. Phosphorus increased with age (week effect, p<0.001) and showed both a significant TM group effect (p<0.05) and TM group×week interaction (p<0.05). Magnesium also showed a significant week effect (p<0.01) and TM group×week interaction (p<0.05).

## DISCUSSION

Enteric methane in ruminants is produced exclusively by methanogenic archaea as a byproduct of ruminal microbial fermentation, with methanogens functioning in close association with other fermentative microbes. In the mature rumen, methanogens play a key role in maintaining low hydrogen partial pressures, thereby favoring efficient fermentation. However, knowledge of their establishment and associations with bacterial taxa during calf rumen development remains limited. In this study, we examined rumen fluid samples from early-stage calves to investigate the community composition of methanogenic archaea and their potential associations with bacterial taxa, blood biochemical parameters, rumen fermentation characteristics, and feed intake. Because rumen fluid was collected once at the end of the experiment, the present findings should be interpreted as endpoint associations rather than longitudinal trajectories of methanogen colonization.

Methanogens begin to colonize and become established in calves during the transition from milk feeding to solid feed intake. To better understand this pattern, we provided starter and hay *ad libitum* to track voluntary intake. Growth performance metrics (body weight, wither height, heart girth, abdominal girth, and ADG) did not differ significantly between the high TM and low TM groups at any age, despite greater starter intake in the high TM group. Greater starter intake is expected to increase propionate production, since starch fermentation yields higher proportions of propionate [18]. Propionate is one of the major fermentation products of rumen microorganisms derived from carbohydrates and serves as the primary substrate for gluconeogenesis [19]. In addition, a positive relationship between the molar proportion of propionate and growth in ruminants has been reported [20]. However, the low TM group exhibited a higher molar proportion of propionate despite lower starter intake than the high TM group, indicating a distinct rumen fermentation pattern between groups.

Higher concentrations of dissolved hydrogen have been associated with greater numbers of methanogens [21]. Furthermore, propionate formation competes with methanogenesis for metabolic hydrogen in the rumen [22]. Higher propionate production in the low TM group may have been accompanied by relatively lower methanogen abundance. However, total methanogen abundance does not directly represent methane emissions, because methane production is also affected by methanogen activity, substrate availability, hydrogen availability, fermentation pathways, and rumen passage dynamics [23]. Therefore, the higher propionate proportion and lower total methanogen abundance observed in the low TM group should be interpreted as a fermentation pattern associated with lower methanogen abundance, rather than as direct evidence of reduced methane production or improved energy efficiency.

Differences in the pattern of starter intake may also have been associated with endpoint differences in total methanogen abundance. The low TM group showed a slower increase in starter intake compared with the high TM group, with markedly lower intake at weeks 6 and 7. Moreover, starter intake at week 6 was positively correlated with total methanogen abundance measured at the end of the experiment. Consistent with our observations, Hennessy et al. [24] reported that calves fed starter feed ad libitum exhibited decreased milk intake and increased starter consumption around week 6. Similarly, Indugu et al. [25] demonstrated that the pre-weaning transition period at 6–8 weeks was accompanied by a marked increase in starter intake and coincided with a major shift in methanogenic archaeal communities. Although differences in breed, diet composition, and sampling strategy exist between studies, these findings support the biological relevance of starter intake during the weaning transition. However, because rumen fluid was collected only once in the present study, the observed association between week 6 starter intake and total methanogen abundance should be interpreted as an end-of-experiment association rather than evidence of longitudinal changes in ruminal methanogen colonization. Future studies with repeated rumen sampling are needed to clarify whether starter intake patterns during this transition are related to temporal changes in methanogen establishment and rumen microbial development.

As solid feed is introduced into a calf’s diet, fermentation activity in the rumen increases and is accompanied by shifts in microbial composition. Previous studies have reported that the transition from the pre-weaning to post-weaning stage is generally accompanied by an increase in Firmicutes and a decrease in Bacteroidota, resulting in a higher F/B ratio [26,27]. Members of Bacteroidota have a strong capacity to degrade proteins and polysaccharides [28], and some taxa within this phylum may contribute to hydrogen utilization in the rumen ecosystem [29]. In contrast, some members of Firmicutes include fibrolytic and carbohydrate-fermenting bacteria that may contribute to hydrogen production during ruminal fermentation [30]. Although previous rumen studies have reported that changes in the F/B ratio accompanied shifts in hydrogen flow under inhibited methanogenesis [31], this ratio should be interpreted cautiously. Hydrogen production and utilization are distributed across diverse rumen microorganisms belonging to multiple phyla and are mediated by various metabolic pathways; therefore, ruminal hydrogen dynamics should not be inferred solely from the relative abundance of Firmicutes and Bacteroidota [32]. In addition, the F/B ratio may not be readily generalized across animal studies because it can be affected by sampling, sample processing, inter-individual variation, diet composition, and feed additives [33]. In the present study, the relative abundance of Firmicutes was higher in the high TM group, whereas that of Bacteroidota was higher in the low TM group. Therefore, the F/B ratio was interpreted only as a descriptive indicator of broad microbial community shifts associated with total methanogen abundance, not as a direct proxy for hydrogen dynamics, methanogen establishment, or methane production.

*Methanobrevibacter* is the dominant methanogen in the rumen [34] and is involved in hydrogenotrophic methanogenesis by using CO_2_ and H_2_ to produce CH_4_. *Methanobrevibacter* was significantly enriched in the high TM group, consistent with the higher total methanogen abundance observed in this group. Eubacterium is an important fiber-degrading genus in the rumen, with roles in hemicellulose breakdown, and is also known to produce butyrate [35]. Because Eubacterium spp. are well-characterized hydrogen producers [36], their higher abundance in the high TM group suggests a possible association with greater hydrogen availability and the tendency toward higher ruminal butyrate proportion observed in this group. *Olsenella* is a lactic acid-producing bacterium that ferments starch and glycogen to produce lactic, acetic, and formic acids [37]. In the present study, *Olsenella* tended to be more abundant in the high TM group, which may be related to greater starter intake and starch fermentation.

*Prevotella* and *Succinivibrio* can produce propionate as a major fermentation product, as reported in other rumen studies [38]. *Prevotella* is one of the most abundant and metabolically versatile bacterial genera in the rumen, known for its ability to degrade a wide range of proteins and polysaccharides. Numerous studies have reported that a higher abundance of *Prevotella* is often associated with reduced methane emissions and increased propionate production [39]. *Succinivibrio* also diverts hydrogen toward succinate and ultimately propionate production [40], thereby making less hydrogen available for methanogenesis. *Selenomonas* also produces propionate through the succinate pathway [41]. Additionally, these bacteria are capable of degrading proteins and are considered hydrogenophilic, primarily participating in fumarate and nitrate reduction pathways within the rumen [42]. By utilizing hydrogen, they compete with methanogens, and their increased abundance has been associated with lower methane emissions in ruminants [43]. In our results, these genera-*Prevotella*, *Succinivibrio*, and *Selenomonas-*were more abundant in the low TM group and showed negative correlations with methanogen abundance and positive correlations with propionate concentrations. Through their ability to divert hydrogen toward alternative fermentation pathways such as the succinate–propionate pathway, these genera may be associated with a propionate-oriented fermentation profile and lower total methanogen abundance. Thus, these taxa may represent microbial features associated with low ruminal total methanogen abundance in calves. However, their relationship with actual methane emissions requires direct validation.

Although the *Clostridiales vadinBB60 group* has been considered a short-chain fatty acid (SCFA)-producing taxon [44], its functional roles remain largely unclear. This taxon has been reported to correlate with propionate production in cattle [45]. Therefore, its higher abundance in the low TM group may be related to the higher propionate proportion observed in this group, although this association should be interpreted cautiously because its functional role remains poorly characterized.

The present results indicate that differences in total methanogen abundance were associated with only limited changes in systemic blood biochemical profiles during early calf development, whereas most biochemical parameters and enzyme activities were mainly age-related, consistent with the age-associated changes in serum metabolites previously reported in Qinchuan cattle [9]. This is consistent with the rapid physiological transition occurring in the first weeks of life, when increasing solid feed intake stimulates rumen fermentation, VFA production, and ruminal epithelial development, leading to large developmental shifts in host metabolism [46]. Albumin was the most consistent between-group difference, being lower in calves with high total methanogen abundance. Albumin is often regarded as a negative acute-phase reactant and can be influenced by inflammatory status, hepatic protein synthesis, and longer-term nutritional adequacy [47]. However, because inflammatory markers, passive transfer indicators, and detailed nutrient intake data were not available in the present study, the biological meaning of this albumin difference remains unclear. Significant TM group×week interactions for cholesterol, phosphorus, and magnesium suggest that some group differences varied depending on age. Such transient patterns are plausible during the 4–8 week transition, when rumen function and epithelial transport rapidly mature. In particular, magnesium homeostasis is known to shift with rumen development, as Mg absorption transitions toward the rumen after the dietary change from milk to solid feed [48], which could contribute to week-dependent group differences. Overall, these results indicate that blood biochemical profiles were influenced mainly by developmental stage, and group-related differences should be interpreted cautiously.

The present study has several main limitations. First, time-series sampling of rumen fluid was not performed. Rumen fluid was collected only once at the end of the experiment; therefore, the present results reflect end-of-experiment differences in rumen microbial and fermentation profiles rather than longitudinal trajectories of methanogen colonization. Although rumen fluid sampling at very early stages may require euthanasia [49] and repeated oral rumen fluid collection in very young calves is technically challenging, future studies should develop and validate safe repeated-sampling or non-invasive approaches to monitor early rumen microbial establishment.

Second, the high and low TM groups were retrospectively defined from the upper and lower quartiles of total methanogen abundance, resulting in a small number of animals per group (n = 6). This approach increased the contrast in methanogen abundance but limited statistical power and the robustness of microbiome comparisons. Therefore, the differentially abundant taxa identified in this study should be interpreted as exploratory and require validation in larger longitudinal cohorts.

Third, although total methanogen abundance was used as a proxy indicator, direct measurement of methane emissions was not conducted. Accordingly, total methanogen abundance should be interpreted as an indicator of methanogen population size, not as a direct measure of methane production. Methane production can also be influenced by methanogen activity, substrate availability, hydrogen availability, rumen fermentation pathways, and passage dynamics. Simultaneous methane quantification would strengthen the validation of the total methanogen qPCR-based approach and provide a more robust basis for interpreting associations between methanogen abundance and host metabolic traits.

## CONCLUSION

In conclusion, calves retrospectively classified by ruminal total methanogen abundance showed distinct rumen fermentation and microbial profiles. The low TM group showed a higher ruminal propionate proportion and greater abundance of several propionate-associated bacterial genera, including *Prevotella*, *Succinivibrio*, and *Selenomonas*. In contrast, systemic blood biochemical parameters were largely driven by age rather than methanogen status. These findings suggest that rumen bacterial composition and fermentation characteristics are associated with ruminal total methanogen abundance in calves. Further longitudinal studies with repeated rumen sampling and direct methane measurement are needed to validate the relationship between early-life feeding patterns, methanogen abundance, and methane-related traits.

## Figures and Tables

**Table 1:** Ingredient and nutrient composition of milk replacers, starter and bermudagrass hay used in this study

Nutrient composition (%)	Milk replacer1)	Bermudagrass hay2)	Starter feed3)
Total digestible nutrients	98		75
Crude protein	23	8.15	22.5
Crude fat	11.5	1.20	2.0
Crude fiber	1.0		10
Ash	10	6.86	10
Calcium	0.6		0.7
Phosphorus	0.4		0.4

1) 77% Skimmed milk powder, concentrated whey protein, dried whey, 16% specific animal fat, vegetable fat, lecithin, salt, casein phosphopeptide, glucose, dextran fermentation by-product concentrate, dried yeast cell walls, whole egg powder, oligosaccharides, silica, silicon dioxide, lactic acid bacteria, bifidobacteria, calcium carbonate, calcium phosphate, and 7% enzymatically hydrolyzed wheat gluten.

2) Neutral detergent fiber 59.3%, acid detergent fiber 30.2%.

3) 48% Corn, soybean meal, wheat bran, and heat-treated corn; 29% soybean oil residue; 16% bran, corn gluten, and rice bran; 7% alfalfa hay cubes, molasses, calcium carbonate, salt, liquor ice extract, and stevia.

**Table 2:** Comparison of growth performance quantification of total methanogen and bacteria, and rumen fermentation characteristics between High and Low TM group

Variables	High TM	Low TM	SEM	p-value
Growth performance				
Daily gain (kg/d)	0.64	0.69	0.03	0.435
Weaning (d)	60.67	60.83	0.81	0.925
Quantification of rumen microbiota (log copy number/ng DNA)				
Total methanogen	6.56	5.53	0.16	<0.0001
Total bacteria	7.20	7.17	0.02	0.359
Rumen fermentation characteristics				
pH	6.52	6.80	0.09	0.136
Ammonia (ng/mL)	10.70	7.60	1.15	0.204
Total VFA (mM)	75.30	73.38	4.72	0.851
Acetate (%)	62.05	59.58	1.49	0.434
Propionate (%)	20.58	28.59	2.09	<0.05
Acetate/propionate	3.08	1.90	0.47	0.241
Butyrate (%)	12.29	7.61	1.32	<0.1
Isobutyrate (%)	0.64	0.82	0.09	0.324
Valerate (%)	3.73	2.59	0.40	0.165
Isovalerate (%)	0.70	0.81	0.09	0.579

Calves were classified into the High TM and Low TM groups based on ruminal total methanogen abundance.

High TM, calves with high ruminal total methanogen abundance; Low TM, calves with low ruminal total methanogen abundance; SEM, standard error of the mean; VFA, volatile fatty acids.

## SUPPLEMENTARY MATERIAL

**Supplement 1.** Changes in blood biochemical parameters related to liver function, electrolyte balance, and mineral metabolism in calves with high and low ruminal total methanogen abundance during the experimental period.
