# Supplementary material for: Ruminal methanogen abundance and associated physiological characteristics in Japanese Black–Holstein F1 calves
Source: Anim Biosci. 2026 Jul 27;39(9):260205. doi: 10.5713/ab.260205 (PMC13547609; doi:10.5713/ab.260205)

Supplement 1. Changes in blood biochemical parameters related to liver function, electrolyte balance, and mineral metabolism in calves with high and low ruminal total methanogen abundance during the experimental period. Blood biochemical parameters were compared between calves with high ruminal total methanogen abundance (High TM) and low ruminal total methanogen abundance (Low TM). Calves were classified into the High TM and Low TM groups based on ruminal total methanogen abundance. Data are presented as the mean ± SEM. *P*-values indicate the effects of week, TM group (Methane effect), and their interaction. * and # indicate $P < 0.05$ and $0.05 \leq P < 0.10$ between groups at each week, respectively. TM, total methanogen; ALP, alkaline phosphatase; AST, aspartate aminotransferase; ALT, alanine aminotransferase; LD, lactate dehydrogenase; γ-GT, gamma-glutamyl transferase; CK, creatine kinase.

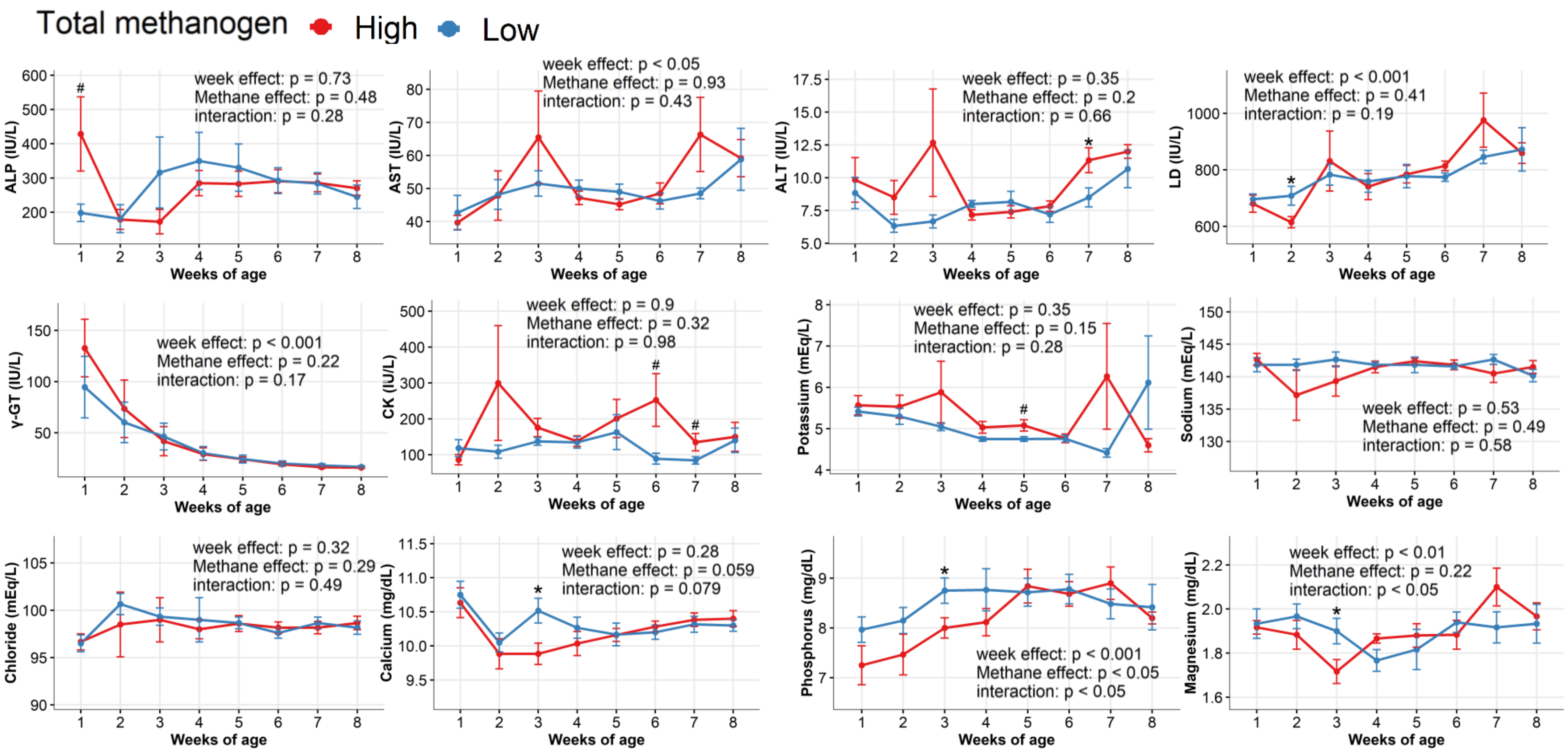

Supplement: Supplementary file 1 [file ab-260205-Supplementary-1.pdf]
